# Allele-Selective Thiomorpholino Antisense Oligonucleotides as a Therapeutic Approach for Fused-in-Sarcoma Amyotrophic Lateral Sclerosis

**DOI:** 10.3390/ijms25158495

**Published:** 2024-08-03

**Authors:** Rita Mejzini, Marvin H. Caruthers, Balazs Schafer, Ondrej Kostov, Kavitha Sudheendran, Marija Ciba, Mathias Danielsen, Steve Wilton, Patrick Anthony Akkari, Loren L. Flynn

**Affiliations:** 1 Centre for Molecular Medicine and Innovative Therapeutics, Health Futures Institute, Murdoch University, Murdoch, WA 6150, Australia; s.wilton@murdoch.edu.au (S.W.); a.akkari@murdoch.edu.au (P.A.A.); loren.flynn@murdoch.edu.au (L.L.F.); 2 The Perron Institute for Neurological and Translational Science, Nedlands, WA 6009, Australia; 3 Department of Biochemistry, University of Colorado, Boulder, CO 80309, USA; marvin.caruthers@colorado.edu (M.H.C.); balazs.schafer@colorado.edu (B.S.); ondrej.kostov@colorado.edu (O.K.); kavitha.sudheendran@colorado.edu (K.S.); marija.ciba@colorado.edu (M.C.); mathias.danielsen@colorado.edu (M.D.); 4 Centre for Neuromuscular and Neurological Disorders, University of Western Australia, Crawley, WA 6009, Australia; 5 Black Swan Pharmaceuticals, Wake Forest, NC 27587, USA; 6 Department of Neurology, Duke University, Durham, NC 27708, USA

**Keywords:** FUS, antisense oligonucleotides, allele-selective, thiomorpholino, amyotrophic lateral sclerosis

## Abstract

Pathogenic variations in the fused in sarcoma (*FUS*) gene are associated with rare and aggressive forms of amyotrophic lateral sclerosis (ALS). As FUS-ALS is a dominant disease, a targeted, allele-selective approach to *FUS* knockdown is most suitable. Antisense oligonucleotides (AOs) are a promising therapeutic platform for treating such diseases. In this study, we have explored the potential for allele-selective knockdown of *FUS*. Gapmer-type AOs targeted to two common neutral polymorphisms in *FUS* were designed and evaluated in human fibroblasts. AOs had either methoxyethyl (MOE) or thiomorpholino (TMO) modifications. We found that the TMO modification improved allele selectivity and efficacy for the lead sequences when compared to the MOE counterparts. After TMO-modified gapmer knockdown of the target allele, up to 93% of FUS transcripts detected were from the non-target allele. Compared to MOE-modified AOs, the TMO-modified AOs also demonstrated reduced formation of structured nuclear inclusions and SFPQ aggregation that can be triggered by phosphorothioate-containing AOs. How overall length and gap length of the TMO-modified AOs affected allele selectivity, efficiency and off-target gene knockdown was also evaluated. We have shown that allele-selective knockdown of FUS may be a viable therapeutic strategy for treating FUS-ALS and demonstrated the benefits of the TMO modification for allele-selective applications.

## 1. Introduction

Amyotrophic lateral sclerosis (ALS) is a fatal motor neuron disease characterized by degenerative changes in both upper and lower motor neurons [1]. Up to 10% of ALS cases have at least one other affected family member and are defined as having familial ALS (fALS) [2]. Fused in sarcoma (FUS) is an RNA-binding protein that is associated with aggressive and early onset ALS cases [3,4,5,6]. Over 50 autosomal dominant pathogenic *FUS* variants have now been identified in ALS patients, accounting for 3–6% of fALS cases identified to date [7]. The majority are missense mutations, although in rare cases, insertions, deletions, and splicing and nonsense mutations have been reported [8]. Many of the pathogenic variants are clustered within the nuclear localization signal and lead to the redistribution of FUS to the cytoplasm [9,10]. Other mutations occur in the glycine- and arginine-rich regions, the low-complexity prion-like domain, and the 3′ UTR [11]. Variants within some regions appear to increase the propensity of the protein to form solid aggregates, pointing to various pathomechanisms operating in FUS-related ALS [12].

Antisense oligonucleotides (AOs) are nucleic acid analogues that are designed to bind to a target RNA and modulate gene expression through various mechanisms. AOs are emerging as a promising new approach to treating ALS and other neurodegenerative diseases, with AOs targeting several ALS disease-related genes currently in pre-clinical development and undergoing clinical trials [13]. Gapmers are AOs composed of a central core of DNA, “the gap”, which is flanked by chemically modified ribonucleotide “wings”, that protect the core from degradation by nucleases. When a gapmer binds its target, the DNA:RNA duplex formed between the target RNA and the central DNA core acts as a substrate for the endogenous enzyme RNase H. RNase H cleaves the RNA strand leading to the degradation of the transcript and subsequent downregulation of the target gene [14].

A range of chemical modifications, including alternative nucleobase, sugar, and linkage modifications, are routinely used in AO design and have been essential to the success of AO-based medicines. One of the most widely used is the phosphorothioate (PS) linkage modification in which the non-bridging oxygen atom in the phosphate group is substituted with a sulfur atom [15]. This enhances the nuclease resistance of the oligonucleotide and increases its lipophilicity, leading to increased interactions with cell surface, plasma, and intracellular proteins [16]. These interactions promote cellular uptake and subcellular trafficking but can also contribute to increased toxicity and immune activation [16,17].

**Figure 1:**
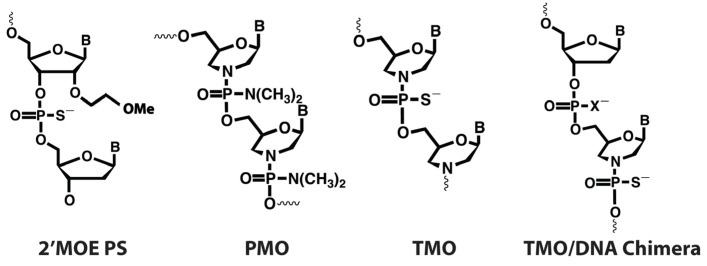
Representation of antisense oligonucleotide chemical structures. X = O or S.

The 2′-methoxyethyl (MOE) sugar modification (Figure 1) is increasingly being utilized in AO drug design, currently featuring in at least six AO drugs that have been approved by the US Food and Drug Administration or the European Medicines Agency, with several more in clinical trials [18]. The MOE modification further improves nuclease resistance of PS oligomers, as well as the affinity and specificity of an AO toward its target and can be used in chimeric antisense designs such as gapmers [19]. Phosphorodiamidate morpholino oligomers (PMOs) are oligonucleotide analogues in which the five-carbon sugar rings are substituted with 6-carbon synthetic morpholine rings that are joined via phosphorodiamidate linkages (Figure 1). PMOs exhibit several advantages over PS AOs, including increased nuclease resistance and enhanced safety with minimal toxicity at clinically relevant doses due to reduced protein binding and reduced immune activation [20,21,22,23]. PMOs, however, are not readily synthesized as chimeras with a DNA core and are therefore not compatible with RNase H activity [20,21]. Additionally, they are currently costlier to produce than PS-based AOs.

Another oligonucleotide analogue, thiophosphoramidate morpholino oligomers (TMOs), has recently been developed [24]. This analogue integrates aspects of both PS oligomers and morpholinos as they are composed of morpholine nucleosides joined by thiophosphoramidate linkages (Figure 1). TMOs provide benefits of both chemistries, including improved cellular uptake, RNA binding affinity and nuclease resistance [24]. Additionally, TMOs can be synthesized on the readily available automated DNA synthesizers. As chimeras with DNA bases, they overcome the cost limitations of PMOs while allowing for their use in RNase H-mediated transcript downregulation [24].

An *FUS*-targeted AO (Ulefnersen) developed by IONIS pharmaceuticals (previously known as ION363) [25] is currently undergoing clinical testing to treat ALS patients with pathogenic variants in *FUS* (NCT04768972). This AO has a gapmer-based design with PS modifications and the widely used 2′-MOE sugar modification in the wings (2′-MOE PS). Ulefnersen reduces expression of all copies of the *FUS* transcript. As FUS-ALS is a dominant disease with the vast majority of patients carrying a pathogenic variant in only one allele, an allele-selective therapy could provide a significant advantage to *FUS* mutation-positive patients. This is because an allele-selective therapy would allow for normal FUS protein expression, minimizing the potential for undesirable effects associated with FUS loss of function. We have developed allele-selective *FUS*-targeted AOs to selectively knock down *FUS* pre-mRNA and mRNA containing a pathogenic variant whilst allowing for expression of the normal allele.

Our allele-selective AOs are-based on the gapmer-based design and are targeted directly to common polymorphisms, relying on this variation between alleles to enable selectivity to the disease target. Several properties of the gapmers can be modified to increase selectivity to the target allele. Modifications can include the number, placement, and base composition of mismatches between the gapmer and the target RNA, as well as changes in oligomer length, gap length, and chemistry. The optimal design of allele-selective gapmers is not easily discernible, as multiple factors can contribute to selectivity/specificity. The effect of AO design modifications, therefore, generally needs to be determined experimentally.

**Figure 2:**
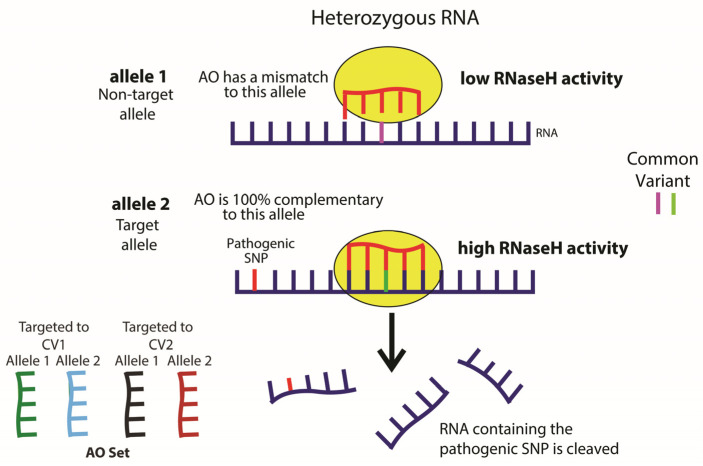
Schematic of allele-selective therapeutic strategy. An AO is selected for the patient that is perfectly complementary to one allele at the site of a heterozygous common variant that is on the same allele as their pathogenic variant. This leads to transcripts from this allele being selectively degraded by RNase H.

Given the large number of pathogenic *FUS* variants, each variant is exceedingly rare, making it technically challenging to develop efficient mutation specific treatments for each. To overcome this, our AOs have been designed to target common variants (CVs) in the *FUS* gene for which a large proportion of FUS-ALS patients are heterozygous. The benefit of this approach is that only a handful of AOs are needed to treat the majority of FUS-ALS patients, irrespective of which pathogenic variant they harbor (Figure 2).

In this study, we demonstrate that allele-selective *FUS* knockdown is achievable using TMO-modified gapmers where the TMO modifications are in the wings. Using this type of construct, allele selectivity increases and SFPQ aggregation is reduced in cultured cells when compared to AOs with MOE-modified wings. We also evaluated how altering total AO length and gap length of TMO-modified gapmers affects allele selectivity, activity, and the hybridization-dependent knockdown of off-target genes.

## 2. Results

### 2.1. FUS Common Variant Analysis

Common variants in *FUS* were analyzed using data from the 1000 Genomes Project [26]. Two exonic common variants were selected as AO target sites. The first, rs741810 (CV1), is a synonymous C/A variation in exon 3. The second, rs1052352 (CV2), is a synonymous C/T variation in exon 4. The genotypes for each of the individuals in the 1000 genome dataset were analyzed to determine the proportion of people in each population that were heterozygous at one or both of the selected *FUS* CVs. All populations had a higher proportion of people heterozygous for CV2 than CV1. The variants are in high linkage disequilibrium; however, targeting both CV1 and CV2 increases the proportion of the populations that are heterozygous for at least one variant by up to 10.6% (Table 1).

### 2.2. Allele Selectivity of MOE and TMO-Modified FUS-Targeted AOs

As the 2′-MOE sugar modification is one of the most widely used for transcript degrading antisense applications, we directly compared AOs with MOE- or TMO-modified wings targeted to *FUS* CV1 and CV2. The AOs were 20 bases in length, with a 10-base gap (5-10-5). All AOs had the PS modification throughout. The AO sequences and chemistry of all AOs used in this study can be seen in Table 2. Based on preliminary work, the CV binding site was placed toward the center of the gap for the CV2 targeted AOs and the CV1 allele 1 targeted AO. Two AO sequences were compared that target CV1 allele 2 with the placement of the CV binding site in the center and toward the 5′ end of the gap.

AOs were transfected at two concentrations (25 nM and 10 nM) into human fibroblasts heterozygous for both CV1 and CV2 (allele 1: CV1 A, CV2 C, allele 2: CV1 C, CV2 T). Controls included a scrambled AO sequence not predicted to bind any human RNA, selectivity control AOs that were targeted to a region in *FUS* with no variation between alleles, and control cells that were treated only with the transfection reagent. Cells were collected for RNA analysis by the ddPCR assay after 72 h incubation.

**Figure 3:**
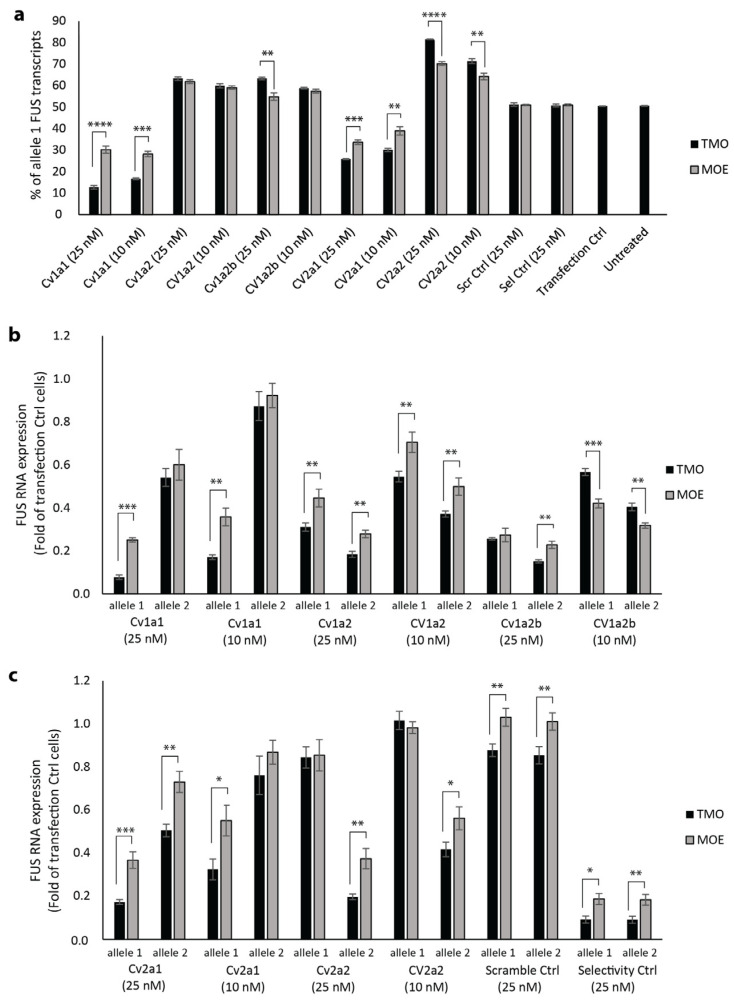
Comparison of allele selectivity of TMO- and MOE-modified *FUS*-targeted AOs. CV1a1 and CV2a1 are targeted to allele 1. CV1a2, CV1a2b, and CV2a2b are targeted to allele 2. AOs were transfected using Lipofectamine 3000 (25 nM and 10 nM) into human fibroblasts heterozygous at CV1 and CV2. RNA was extracted and analyzed by ddPCR after 72 h incubation. (**a**) Percentage of *FUS* allele 1 transcripts. (**b**) *FUS* mRNA expression after transfection with CV1-targeted AOs presented as fold-change compared to levels in control cells treated with the transfection reagent only (Transfection Ctrl), normalized to levels of housekeeping gene *TBP*. (**c**) *FUS* RNA expression after transfection with CV2-targeted AOs. Error bars represent standard error of the mean. *n* = 3. Unpaired Student’s *t*-test. * *p* < 0.1, ** *p* < 0.05, *** *p* < 0.01, **** *p* < 0.001.

For the most selective AOs, CV1a1 (targeted to allele 1) and CV2a2 (targeted to allele 2), reduction in mRNA of the non-target allele was similar after transfection with AOs synthesized using either MOE or TMO chemistry. However, there was significantly greater mRNA reduction of the target alleles after transfection with the TMOs when compared to the MOEs (Figure 3b,c). After a 25 nM transfection with the CV1a1 TMO, allele 1 transcripts accounted for 12.7% of total *FUS* transcripts; this was significantly less than the 30.2% of allele 1 transcripts after transfection with the same AO sequence having the MOE modifications (*p* < 0.001) (Figure 3a). At 25 nM, the CV1a1 TMO reduced expression of the target allele to 0.078-fold that of levels measured in control cells treated only with the transfection reagent, while the non-target allele remained at 0.542-fold of control levels (Figure 3b). At 10 nM, the CV1a1 TMO reduced expression of allele 1 to 0.171-fold with allele 2 remaining at 0.874-fold that of control levels (Figure 3b). After a 25 nM transfection with the CV2a2 TMO, 18.6% of total *FUS* transcripts were transcribed from allele 2 with 81.4% of transcripts from allele 1; this was significantly higher than the 70.2% of allele 1 transcripts after transfection with the same AO sequence having MOE modifications (*p* < 0.001) (Figure 3a). The CV2a2 TMO (25 nM) reduced expression of allele 2 to 0.196-fold control levels, while allele 1 remained at 0.844-fold (Figure 3c).

The remaining AOs (CV1a2, CV1a2b, CV2a1) were less selective than those discussed above, and the chemistry made less of a difference to selectivity (Figure 3a). There was no significant difference in the percentage of allele 1 transcripts after transfection with the CV1a2 TMO compared to the MOE. For the CV1a2b sequence with the SNP at the second position of the gap from the 5′ end, the TMO was more selective than the MOE at the 25 nM concentration. The CV2a1 TMO was more allele-selective than the MOE. For these three AO sequences, the TMO modification increased efficiency, with a larger reduction in expression of both *FUS* alleles compared to the MOE-modified AOs (Figure 3b,c).

### 2.3. TMO Reduces SFPQ Positive Protein Aggregation

**Figure 4:**
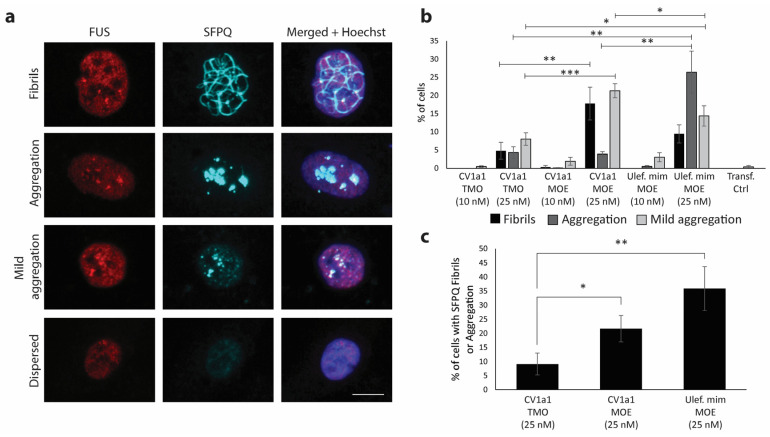
Immunocytochemistry in human fibroblasts. (**a**) Representative images of FUS and SFPQ immunostaining showing cells with SFPQ fibrils, aggregation, mild aggregation, or dispersed SFPQ. Scale bar = 10 µm. (**b**) The mean percentage of human fibroblasts displaying SFPQ fibrils, prominent aggregation, or mild aggregation 24 h after transfection with *FUS*-targeted AO gapmer CV1a1 with TMO- or MOE-modified wings or the Ulefnersen mimic AO (Ulef. mim) at 10 nM and 25 nM (*n* = 5). (**c**) Mean percentage of cells with prominent SFPQ aggregation or fibrils after 24 h (25 nM) (*n* = 5). Approx. 200 cells counted per experiment. Error bars represent standard error of the mean. Unpaired Student’s *t*-test. * *p* < 0.1, ** *p* < 0.05, *** *p* < 0.01.

AOs with a PS backbone have been found to have backbone-specific effects on the altered distribution of nuclear proteins and are associated with the formation of toxic novel structured nuclear inclusions/aggregates that are immunopositive for paraspeckle-associated proteins, including SFPQ [27]. We used immunocytochemistry to examine FUS and SFPQ localization and aggregation in human fibroblasts 24 h after transfection with the CV1a1 AO synthesized with either TMO- or MOE-modified wings. Examples of the different FUS and SFPQ immunostaining patterns observed are shown in Figure 4a. We also tested an AO with the same sequence and chemistry as Ulefnersen as published (Ulefnersen mimic) [25].

The transfection control cells did not display any SFPQ-positive fibrils or prominent SFPQ or FUS aggregation, with only 0.37% of cells showing mild SFPQ aggregation. At the 10 nM transfection concentration, none of the AOs tested led to a significant increase in cells displaying SFPQ fibrils, aggregation, or mild aggregation when compared to the transfection control cells (α = 0.05). At 25 nM, the number of cells with SFPQ-positive fibrils increased to 4.8% for the CV1a1 TMO-treated cells, this was significantly less than the 17.8% seen in the CV1a1 MOE-treated cells (*p* = 0.034). After transfection with the Ulefnersen mimic, 9.48% of cells displayed SFPQ-positive fibrils (Figure 4a,b). The Ulefnersen mimic-treated cells displayed a significantly greater number of cells with prominent non-fibrillar SFPQ aggregation, observed in 26.4% of cells compared to 4.32% in the TMO-treated cells (*p* = 0.017). The CV1a1 MOE-treated cells had a similar number of cells with prominent non-fibrillar SFPQ aggregation as the TMO-treated cells at 3.89% (Figure 4a,b). There were also significantly more cells with mild SFPQ aggregation for both the CV1a1 MOE-treated cells and the Ulefnersen mimic-treated cells compared to the TMO-treated cells (Figure 4a,b). We also compared the number of cells with either SFPQ fibrils or prominent aggregation after a 25 nM transfection, and this was 9.1% for the CV1a1 TMO-treated cells. This increased to 21.7% for the CV1a1 MOE-treated cells (*p* = 0.073) and to 35.9% for the Ulefnersen mimic-treated cells (*p* = 0.015) (Figure 4c).

### 2.4. Effect of Allele-Selective TMOs on FUS Protein Expression

**Figure 5:**
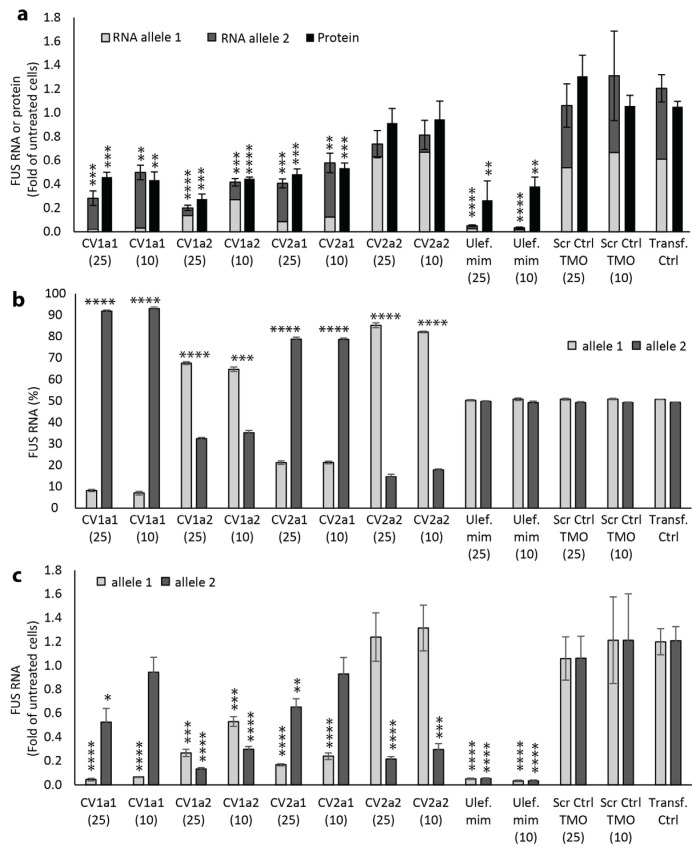
(**a**) FUS mRNA and protein expression five days after transfection with CV1- and CV2-targeted TMOs. FUS protein levels measured by Western blot (normalized to total protein) and total *FUS* mRNA levels measured by ddPCR (normalized to housekeeping gene *TBP*) expressed as fold-change compared to levels in untreated cells. Allele 1 and 2 levels shown for RNA taken from (**b**). (**b**) Percentage of *FUS* transcripts of each allele measured by ddPCR analysis. (**c**) *FUS* mRNA expression after transfection presented as fold-change compared to levels in untreated cells normalized to levels of housekeeping gene *TBP*. CV1a1 and CV2a1 are targeted to allele 1. CV1a2 and CV2a2 are targeted to allele 2. Error bars represent standard error of the mean. *n* = 3. Unpaired Student’s *t*-test compared to untreated cells * *p* < 0.1, ** *p* < 0.05, *** *p* < 0.01, **** *p* < 0.001.

TMOs targeted to each CV and allele were again tested in human fibroblasts, this time with a five-day incubation period. Total FUS protein levels, as well as mRNA expression of each allele, were measured. The half-life of FUS in cell culture is approximately 12 h [28]. Therefore, after a five-day incubation, only a negligible amount of the FUS protein that was translated before the transfection period would be expected to be present. Consequently, the measured FUS protein levels were expected to be dependent on the AO treatment. For all AOs tested, the percentage of *FUS* transcripts of the target allele was reduced further than in previous experiments that had only a three-day incubation period. In most cases, there was no significant difference in total FUS protein expression when the AO concentration was increased from 10 nM to 25 nM (Figure 5a). The percentage of each allele also remained similar in each case when AO concentration was increased (Figure 5b).

We found there was a large variation in the total FUS protein levels measured in cells treated with the two lead AOs, CV1a1 targeted to allele 1 and CV2a2 targeted to allele 2. Total FUS protein expression was reduced to 0.429-fold that of levels in untreated cells after a 10 nM transfection with CV1a1 (*p* = 0.014) (Figure 5a). Most of this protein was likely translated from allele 2, which made up 93.1% of the total *FUS* transcripts (Figure 5b). In this case, allele 1 transcripts were at 0.066-fold of the levels in control cells and allele 2 transcripts were at 0.945-fold (Figure 5c). In the CV2a2-treated cells, FUS protein levels were not significantly different from levels in untreated cells at 0.937-fold that of control levels after a 10 nM transfection (Figure 5a). In this case, 82.2% of *FUS* transcripts came from allele 1 (Figure 5b). Levels of allele 2 were at 0.296-fold the control levels, while allele 1 levels had increased to 1.32-fold (Figure 5c). This indicates that *FUS* transcript levels were being upregulated in response to the AO, leading to greater FUS expression from the non-target allele.

The greatest difference in FUS protein expression with varying AO concentration was for the CV1a2 AO-treated cells where FUS protein expression was at 0.440-fold and 0.270-fold that of the untreated cells after 10 nM and 25 nM transfections, respectively. For the CV2a1-treated cells (10 nM), FUS protein levels were at 0.479-fold that of control levels with 79% of mRNA transcripts coming from allele 2. Generally, the total FUS mRNA and protein levels were closely aligned. Notably, this was not the case for the cells transfected with the Ulefnersen mimic in which total *FUS* RNA levels were measured at only 0.034- and 0.052-fold that of control levels, while protein levels were at 37.6% and 26.1% of control levels after a 10 nM and 25 nM transfection (Figure 5a,c).

### 2.5. Effect of Reducing Total Length and Gap Length on Efficacy and Selectivity

Additional TMO-modified gapmers targeted to each CV and allele were tested (10 nM) to determine whether allele selectivity or efficiency could be improved. This included AOs that retained the 20-base length but reduced the gap size to 8 or 6 bases, as well as AOs that reduced both the total length and gap length.

**Figure 6:**
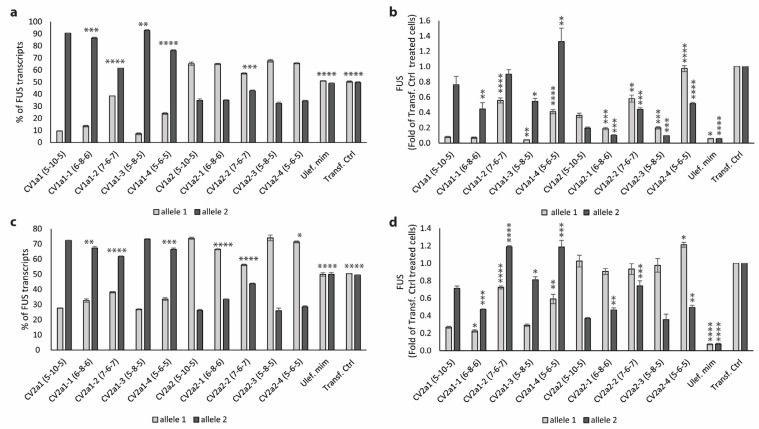
Comparison of selectivity and activity of AOs with differing gap length and/or total length. AOs were transfected using Lipofectamine 3000 (10 nM) into human fibroblasts heterozygous at CV1 and CV2. After 72 h incubation, RNA was extracted and analyzed by ddPCR. (**a**) Percentage of *FUS* transcripts of each allele after transfection with CV1-targeted AOs. (**b**) Expression of each *FUS* allele after transfection with CV1-targeted AOs presented as fold-change compared to levels in control cells treated only with the transfection reagent (Transf. Ctrl) normalized to levels of housekeeping gene *TBP*. (**c**) Percentage of *FUS* transcripts of each allele after transfection with CV2-targeted AOs. (**d**) Expression of each *FUS* allele after transfection with CV2-targeted AOs presented as fold-change compared to levels in control-treated cells. Error bars represent standard error of the mean. *n* = 3. Unpaired Student’s *t*-test comparing AOs to the original (5-10-5) AO designs. For Ulef. mim., allele 1 is compared to the allele 1 targeting AO and allele 2 to the allele 2 targeting AO. * *p* < 0.1, ** *p* < 0.05, *** *p* < 0.01, **** *p* < 0.001.

For the AOs targeted to allele 1 at CV1, reducing the length of the gap from 10 to 8 bases while retaining overall length (6-8-6), led to reduced allele selectivity with the proportion of transcripts measured from the target allele increasing from 9.55% to 13.65% (*p* < 0.01) (Figure 6a). Expression of the target allele was similar at 0.079- and 0.070-fold of control levels, while expression of the non-target allele was reduced significantly from 0.764-fold to 0.446-fold that of control levels (*p* = 0.043) with the reduced gap length (Figure 6b). In contrast, when the gap length was reduced to eight bases and the overall AO length was also reduced (5-8-5), allele selectivity was improved slightly compared to the original 5-10-5 design with the proportion of transcripts from the target allele reduced from 9.55% to 7.24% (*p* = 0.031). AO activity also increased, with expression of the target allele reduced to 0.042-fold and the non-target allele to 0.54-fold of control levels (Figure 6b). Further reducing the gap length to six bases while retaining overall AO length (7-6-7) greatly reduced allele selectivity with 38.5% of *FUS* transcripts now expressed from the target allele (*p* < 0.001). AO activity was also significantly reduced compared to the original (5-10-5) AO design with expression of the target allele increased to 0.557-fold of control levels (*p* < 0.001). The AO with the six-base gap performed better when overall AO length was also reduced (5-6-5) but was still much less effective than the original 5-10-5 AO design (Figure 6a,b).

For AOs targeted to allele 2 of CV1, the percentage of transcripts from each allele was similar for all AOs tested with only the (7-6-7) design leading to an increased proportion of the target allele (*p* = 0.0052). There were, however, differences in AO activity, with the eight-base gap length (6-8-6) and (5-8-5) increasing AO activity slightly and the six-base gap length (7-6-7) and (5-6-5) reducing the activity substantially (Figure 6a,b).

A similar result was seen with the CV2-targeted AOs. For the AOs targeted to allele 2, allele selectivity was again reduced by reducing the size of the gap from 10 to 8 (6-8-6) with the proportion of transcripts from the target allele increasing significantly from 26.2% to 33.5% of total *FUS* transcripts (*p* = 0.001) (Figure 6c). Compared to the (5-10-5) AO design, total expression of the target allele increased slightly from 0.369-fold control levels to 0.465-fold (*p* = 0.027), while expression of the non-target allele was reduced from 1.02- to 0.907-fold (*p* = 0.191) (Figure 6d). When the gap size was reduced to eight bases with the total AO length also reduced (5-8-5), allele selectivity and activity did not differ from the original (5-10-5) AO design. Reducing the gap size to six while retaining the 20-base total length (7-6-7) again greatly reduced the selectivity and activity of the AO. When total AO length was also reduced (5-6-5), selectivity and AO activity were improved but were not as effective as the original 5-10-5 AO design. For the AOs targeted to allele 1 at CV2, again, allele selectivity was reduced slightly by shortening the gap length from 10 to 8 (6-8-6) and improved to levels similar to the original (5-10-5) design by also reducing the total length (5-8-5). Reducing the gap size to six, (7-6-7) and (5-6-5), again reduced selectivity and AO activity (Figure 6c,d).

### 2.6. Hybridization-Dependent Off-Target Gene Knockdown

AOs have the potential to induce off-target effects by inadvertent binding to unintended RNAs that have sequence similarity to the target RNA. When designing AOs for therapeutic use, it is important to consider and manage this possibility at the design stage. Changes in AO length can have bilateral effects on hybridization-dependent changes in the expression of non-target genes. Increasing AO length reduces the number of off-target candidate genes with perfect or near-perfect matches to the target; however, increased length also increases the binding affinity between the AO and complementary RNA, leading to better tolerability of mismatches [29]. The size of the “gap” that allows for RNase H activity may also impact any effect on the downregulation of off-target gene expression.

**Figure 7:**
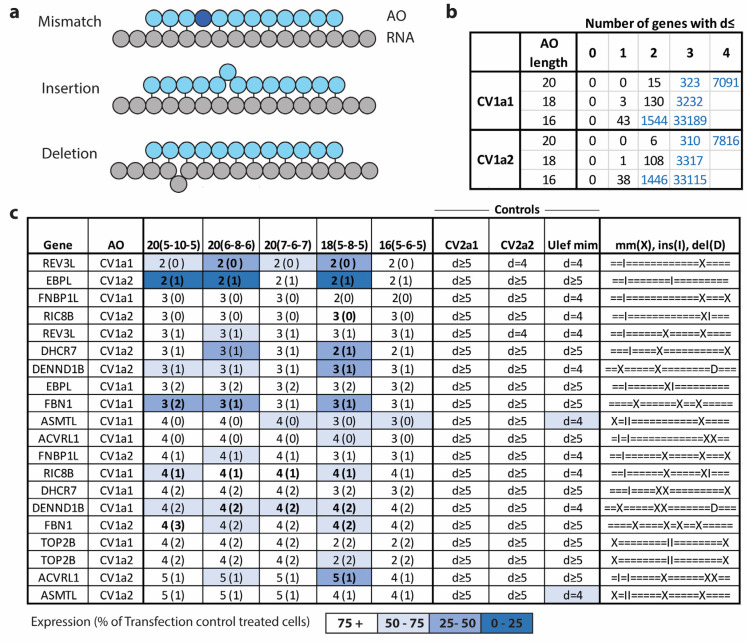
Expression of off-target genes with sequence similarity to the *FUS* CV1 target region. (**a**) Schematic showing alignments between AOs and RNA. (**b**) Table of the number of genes with sequence similarity to the *FUS* target regions with *d* ≤ 4. *d* is the total number of mismatches, insertions, and deletions. Black numbers are the number of unique genes with similar transcripts counted through search results using the online GGGenome tool “https://gggenome.dbcls.jp/ (accessed on 25 January 2023)”. Human pre-spliced RNA (RefSeq curated on GRCh38/hg38.p13, D3G 22.02 (February, 2022), and RefSeq human RNA release 210 (January, 2022)), was analyzed. Blue numbers are estimates based on the counted results. (**c**) Mean expression of select genes with sequence similarity to the *FUS* CV1 AO target region 72 h after transfection with the CV- targeted TMOs with differing gap lengths and total lengths or controls. Measured by qPCR. *n* = 3. Bold indicates that levels were significantly lower than in transfection control-treated cells (α = 0.05). Mismatches (X), insertions (I), and deletions (D) shown in the final column are for the 20-mer AO length with the 18-mers and 16-mers having one or two bases removed from each side.

An analysis of hybridization-dependent off-target effects of selected genes with sequence similarity to the target sequence of the CV1-targeted AOs was undertaken. An in silico analysis identified RNA transcripts with sequence similarity to the target regions of the *FUS* CV1-targeted AOs. Transcripts with four or fewer mismatches, deletions, or insertions (*d*) (Figure 7a) between an AO and the complementary RNA sequence were identified by searching a database of human pre-spliced and spliced RNA using the online search algorithm GGGenome “https://gggenome.dbcls.jp/ (accessed on 25 January 2023)”. For the CV1a1 and CV1a2 sequences, there were no other transcripts that were completely complementary to the 20, 18, or 16-mer AOs. For the 20-mers, there were again no transcripts where *d* = 1. For the 18-mers, there were three genes with *d* = 1 for CV1a1 and one gene for CV1a2. When the AO length is reduced to 16, the number of *d* = 1 genes increased to 43 and 38 for CV1a1 and CV1a2. The number of *d* ≤ 2 genes increased to 15 and 6 for the 20-mers. The number of genes with *d* ≤ 2 increases greatly with decreasing AO length. The number of unique genes with similar transcripts with values of *d* from 0 to 4 for the CV1a1 and CV1a2 AOs with differing lengths can be seen in Figure 7b.

A number of genes were selected for expression analysis where *d* was equal to two, three, four, or five. Genes were selected that varied in the numbers of variations that would be in the area that the gap region of the AO binds. Genes were selected that only had one region where *d* ≤ 4 and had no *d* ≤ 4 regions for at least one of the *FUS* targeted control AOs that were used (CV2a1, CV2a2, or Ulefnersen mimic) so that reduced expression could be attributed to AO binding and RNase H activity rather than as a secondary consequence of reduced FUS expression. Selected genes also needed to be expressed in the cell type being tested, fibroblasts. Expression levels of the selected genes were determined by qPCR after transfection with the CV1 series TMOs with differing length and gap lengths. Results can be seen in Figure 7c.

The AOs with the six-base gap regions (7-6-7) and (5-6-5) caused the least knockdown of the off-target genes. These AOs, however, were also comparably ineffective at reducing expression of the *FUS* target gene. The AOs with the 6-8-6 design led to increased off-target gene knockdown compared to the 5-10-5 design with several genes showing a greater reduction in expression despite having the same number of mismatches or insertions in the gap binding region. These AOs were also less selective when targeted to *FUS* with reduced expression of the non-target allele compared to the 5-10-5 design. The AOs with the (5-8-5) AO design were reasonably comparable in selectivity and activity toward the *FUS* target, with only small increases in activity or selectivity compared to the 5-10-5 design for some AOs. The 5-8-5 AOs, however, led to more off-target gene knockdown for several of the genes tested. In some cases, the reduction in AO length also reduced the number of *d,* while in other cases it remained the same. The AOs with the 5-10-5 AO design caused no or only a minimal reduction in expression of the off-target genes when *d* ≥ 3, with the exception of FBN1, with this design showing the greatest differential between on- and off-target gene silencing. One of the two genes tested where *d* = 2 (EBPL) was reduced quite substantially. Full qPCR results can be found in Supplementary Tables S1 and S2.

## 3. Discussion

Mutations in the *FUS* gene are associated with aggressive forms of ALS that disproportionately affect younger patients. There is strong evidence that many pathogenic *FUS* variants lead to a toxic gain of function of the FUS protein [30,31,32]. Although some reports have suggested that loss of FUS function may not contribute to motor neuron degeneration in ALS, there is evidence that FUS loss of function may also contribute to neurodegeneration through several distinct mechanisms [33,34].

FUS plays a role in several aspects of gene expression, including transcription, pre-mRNA splicing, mRNA transport, and translation regulation [35]. Depletion of FUS from the adult nervous system has been found to alter the levels or splicing of more than 950 mRNAs, including many involved in neuronal function and motor neuron survival [36,37,38]. Notably, loss of FUS leads to the pathological mis-splicing of microtubule associated protein tau (*MAPT*), leading to increased expression of 4R-Tau [39,40]. Increased or aggregated 4R-Tau is associated with several neurodegenerative diseases [41,42].

In addition to gene and splicing regulation, FUS is also involved in multiple genome repair pathways [43,44,45,46]. FUS is required for the activation of the DNA damage response and the proper assembly of DNA double-strand-break repair complexes [47]. Loss of nuclear FUS in motor neurons has been found to cause DNA nick ligation defects [48].

FUS also plays a role in cellular stress responses and is involved in the formation of cytoplasmic stress granules [49]. There is evidence that mutant FUS disturbs stress granule dynamics [9,34,50]. The failure of these transient protein and RNA complexes to disassemble when the stress signal is lifted can lead to the pathological persistence of stress granules that may initiate pathological protein aggregation [51]. On the other hand, inhibiting these stress responses by reducing FUS expression may not be beneficial as they act as a protective mechanism against proteotoxicity [34]. FUS is also an essential component in the formation of paraspeckles [52,53]. Paraspeckles are dynamic nuclear structures that form in response to cellular stressors, affecting gene expression through sequestration of their component proteins. *FUS* mutations promote dysfunctional paraspeckle formation [54]. However, loss of FUS function also disrupts paraspeckle assembly, which may contribute to pathogenesis of FUS-ALS by impairing protective responses in neurons [55].

Antisense-mediated reduction of *FUS* has been shown to effectively suppress FUS expression throughout the CNS, reduce the burden of FUS aggregation, and reverse the insolubility of other RNA-binding proteins in the brain and spinal cord [25]. While this has been an important and exciting development in the pursuit of effective treatments for FUS-ALS patients, reducing normal, as well as toxic, FUS protein expression will not address FUS loss of function. An allele-selective approach to reducing the burden of toxic FUS protein can minimize the effects associated with FUS loss of function, potentially improving the long-term safety of an *FUS*-targeted therapeutic.

We have demonstrated that allele-selective knockdown of *FUS* by the targeting of common SNPs in the gene is a feasible therapeutic strategy for treating FUS-ALS. While the utility of TMO analogues has previously been demonstrated for splice-switching AO applications [56], we now show that TMOs also provide a benefit in allele-selective RNase H-mediated gene knockdown, leading to greater allele selectivity and knockdown of the target alleles when compared to MOE modified AOs.

While modifications to synthetic nucleic acids are required to protect against nuclease degradation and improve drug function, they can also lead to undesired intracellular interactions. AOs that incorporate the PS backbone can bind non-specifically to intracellular proteins to form a variety of toxic inclusions, including highly stable and structured nuclear inclusions that disturb RNA processing and are immunopositive for paraspeckle proteins such as splicing factor SFPQ [27]. Formation of these structured nuclear inclusions was evident after transfection with the fully PS-modified CV1a1 AO, where the MOE modification was used in the wings. Despite the TMO-modified AO also comprising the PS linkage modification in the gap region of the AO and containing a sulfur moiety in the linkage between the nucleotides in the wing regions, we have shown that the TMO modification significantly reduced SFPQ-positive protein aggregations and structured inclusions compared to the MOE modification. Interestingly, the Ulefnersen mimic that contained five fewer PS linkages also led to significantly increased SFPQ aggregation. The formation of structured SFPQ-positive fibrils also increased in the Ulefnersen mimic-treated cells compared to the TMO-modified AO, although this did not reach statistical significance.

We found there was a substantial difference in the allele selectivity of the AOs targeted to each allele for both of the target SNP sites. There are a number of factors that can contribute to the selectivity/specificity of any particular AO. This includes SNP-sensitive differences in secondary structure of the RNA, differences in free energy of AO hybridization to each allele, and how a SNP will alter substrate recognition by RNase H [57,58,59]. A single mismatch in a DNA/RNA heteroduplex can result in a several-fold decrease in cleavage efficiency by RNase H [60]. However, this is highly dependent on the nature of the SNP and the surrounding nucleotides [61]. Additionally, a single base change can determine whether a preferred cleavage site is present [62]. Due to the number of factors influencing RNase H-dependent RNA degradation, the selectivity of an AO to its potential targets generally needs to be determined experimentally. The AO targeted to allele 1 of CV1 (CV1a1) showed high allele selectivity. The mismatch to allele 2 in this case is a T-C (pyrimidine transversion). The AO targeted to allele 2 was much less selective; in this case, the mismatch was a G-A, (purine transversion). In the case of the CV2-targeted AOs, CV2a2 was much more selective than CV2a1. In both cases, the mismatches were transductions, a G-U and an A-C.

The two targeted *FUS* CVs are in high linkage disequilibrium with each other. The A allele of CV1 is usually inherited with the C allele of CV2 in European, American, and Asian populations. This means that of the patients amenable to this allele-selective strategy that are heterozygous at both CVs (approximately 57% of amenable patients in European populations, 65% in American, and 94% in Asian populations), the vast majority would be able to receive one of the two lead AOs (CV1a1 or CV2a2, depending upon which allele their pathogenic *FUS* variant is found). Of the remaining amenable patients that are heterozygous in only one of the two CVs, approximately 50% could also be expected to receive one of the lead AOs. This leaves a relatively small number of amenable patients that would only be able to receive the less selective CV1a2 or CV2a1 AOs. However, these less selective AOs could still maintain higher expression of functional FUS over a non-allele-selective *FUS*-targeted therapy, providing a significant advantage to patients with CV1a2 reducing expression of allele 2 to 35.2% of total *FUS* transcripts after 5 days (10 nM) and CV2a1 reducing allele 1 transcripts to 21.3%. We found that when the incubation period was increased from three days to five days, the proportion of the target alleles detected was further reduced for all AOs tested. The AOs have not yet been tested at lower concentrations or longer timepoints to determine whether reduced expression of targeted alleles or increased FUS protein can be improved further. Allele selectivity may also be further improved through design modifications.

We tested the effect of reducing the gap length and the total AO length on allele selectivity of our TMO-modified gapmers. We found that reducing the AO length or gap length from our original 5-10-5 AO design reduced selectivity and AO activity in most cases. Only the 5-8-5 design led to a small increase in AO activity or allele selectivity in some cases. How changes in AO length and gap length will affect selectivity or efficacy can be hard to predict. RNase H1, which is responsible for AO-mediated cleavage of RNA in eukaryotes [63,64], requires a gap size of at least five for cleavage to occur [65]. The cleavage rate of RNase H1 increases with an increase in the length of substrate. This is because more potential cleavage sites become available, increasing the number of productive binding interactions [65]. Increased AO length also increases binding affinity to a target; however, shorter AOs can sometimes be more potent [66,67]. This is a counterintuitive effect that can be attributed to affinity rather than length per se. Affinities that are too high will result in a slow release of enzyme and oligonucleotide, stalling the catalytic cycle and reducing potency [68]. Specificity is also affected by binding affinity. When affinity is increased, the dissociation of mismatched duplexes becomes slower, increasing the rate of cleavage of mismatched targets [69]. For a given target and a given cell type, there will be an optimal affinity for AO potency and selectivity.

The effect of AO design on hybridization-dependent off-target binding is also an important consideration. Binding affinity can affect off-target gene knockdown by affecting whether appreciable amounts of duplex form or through effects on dissociation. Off-target gene knockdown is also influenced by the ability of RNase H to tolerate structural changes induced by duplex mismatches and bulges [70]. The addition of extra mismatches in gapmers designed to target a specific allele can, in some cases, increase differentiation between two alleles. In other cases, this does not occur and the opposite effect can even be seen [70]. The position of the mismatch within the DNA window and in relation to the position of another mismatch can also influence RNase H activity. Although we found that AO activity towards the target gene was improved slightly by reducing the gap and total length by two bases (5-8-5), this also led to increased knockdown of several of the unintended off-target genes measured. Reducing the AO length also greatly increases the total number of genes with sequence similarity to the target sequence. In this case the 5-10-5 AO design gave the greatest differential between on- and off-target gene silencing.

A splice-switching AO approach to *FUS* knockdown could greatly reduce the issue of off-target gene knockdown as it does not rely on RNase H activity. A splice-switching AO would be useful for patients that are not heterozygous at the *FUS* SNPs being targeted. However, this approach would not be allele-selective, reducing expression of all *FUS* transcripts. The reduced off-target gene knockdown, however, could still prove to be beneficial over a non-selective RNase H-dependent strategy. While the unintended knockdown of genes with sequence similarity is a risk that needs to be considered and managed, it is important to note that a drastic reduction in FUS through a non-allele-selective strategy will also affect the expression of many proteins, with FUS depletion altering the expression and splicing of hundreds of genes.

In conclusion, we have demonstrated that the allele-selective TMO gapmers described in this study represent a promising therapeutic strategy that could be useful to treat FUS-ALS patients while minimizing the effects of FUS loss of function. The average age of disease onset for FUS-ALS patients is much lower than for other ALS types at just 35 years [71]. With the long-term effects of FUS loss of function remaining uncertain, an allele-selective therapeutic approach could have significant clinical benefit for these patients compared to a strategy that reduces all FUS expression. While this strategy has not yet been tested in vivo, future studies will involve testing in patient iPSC-derived motor neurons and in animal models to demonstrate efficacy and the effects on FUS gain and loss of function.

## 4. Materials and Methods

### 4.1. Antisense Oliginucleotide Synthesis

The MOE-modified AOs, including the Ulefnersen mimic, were synthesized by SynGenis (Perth, Australia). TMO-modified AOs were synthesized using previously described procedures [24].

### 4.2. Cell Culture and Transfection

The human fibroblasts (Coriell Cell Repositories, GM03652) were maintained at 37 °C and 5% CO_2_ in a humidified incubator in Dulbecco’s Modified Eagle Medium supplemented with 10% foetal bovine serum. Cells were seeded in 24-well plates at 15,000 cells per well for RNA analysis, on glass coverslips in 12-well plates at 30,000 cells per well for immunocytochemistry experiments, and in 6-well plates at 75,000 cells per well for protein analysis. AOs were transfected into cells using the Lipofectamine 3000 transfection reagent (L3K) (ThermoFisher Scientific, Scoresby, VIC, Australia) according to the manufacturer’s protocol and incubated in opti-MEM culture media (ThermoFisher Scientific) at 37 °C for the desired time period before collection.

### 4.3. RNA Extraction

Total RNA was extracted from the cells using the MagMAX™ 96 Total RNA Isolation Kit (Cat#AM1830, ThermoFisher Scientific, Scoresby, VIC, Australia), according to the manufacturer’s protocol. The purity and concentration of RNA was determined using an Implen NanoPhotometer^®^ N120 (Implen, Westlake Village, CA, USA).

### 4.4. ddPCR Analysis

Droplet digital PCR analysis was carried out using the QX200 droplet digital PCR system and the One-Step RT-ddPCR Advanced Kit for Probes (Bio-Rad Laboratories Pty., Ltd., Gladesville, NSW, Australia). Primers amplified *FUS* with the forward primer in exon 4 and the reverse primer crossing the exon 4/5 junction (Fwd: CTATGGAACTCAGTCAACTCC, Rev: GAACTGCTACCGTAACTTCC) with the temperature profile, 50 °C for 50 min, 95 °C for 10 min, followed by 40 cycles of 95 °C for 30 sec and 55 °C for 1 min and finally 98 °C for 10 min. Primers and probes were purchased from Integrated DNA Technologies Australia (Melbourne, VIC, Australia). Quantification of transcripts containing a T at *FUS* CV2 were detected using a DNA probe with a Hex fluorophore and Iowa Black FQ quencher with the sequence CCTCCTATCCTGGCT, where underlined bases indicate a locked nucleic acid modification. Quantification of transcripts containing a C at *FUS* CV2 were detected using a DNA probe with the sequence CTCCTACCCTGGCTA with an FAM fluorophore and Iowa Black FQ quencher. Total *FUS* levels compared to controls were determined by normalizing *FUS* to *TBP* levels. *TBP* was measured by ddPCR assay in a separate well with forward primer TCTTTGCAGTGACCCAGCATCAC, reverse primer CCTAGAGCATCTCCAGCACACTCT, and a DNA probe with the sequence CCAAGGAATTGAGGAA with an FAM fluorophore and Iowa Black FQ quencher. A no template control and control RNA homozygous for each allele at *FUS* CV2 were run to ensure there was no signal from the other allele for each probe. Data analysis was performed using BioRad’s QuantaSoft™ Analysis Pro software version 1.

### 4.5. Protein Isolation and Western Blot

Cell pellets were lysed in 50 μL of Western blot lysis buffer containing 125 mM Tris/HCl pH 6.8, 15% SDS, 10% Glycerol, 1.25 μM PMSF (Sigma-Aldrich, Melbourne, NSW, Australia) and 1× protease inhibitor cocktail (Sigma-Aldrich). Cells were sonicated at 30 mA (4 × 1 s pulses, followed by a brief vortex and 2 more 1 s pulses) to extract protein. Bromophenol blue (0.004%) and dithiothreitol (50 mM) were added to each sample. Samples were heated on a heat block at 95 °C for 5 min, frozen, thawed, and centrifuged at 14,000× *g* for two minutes and the supernatant used for protein analysis. Protein concentration was determined using a PierceTM BCA Protein Assay Kit (ThermoFisher Scientific, Scoresby, VIC, Australia) according to the manufacturer’s instructions.

Seven μg of protein from each sample was loaded onto a NuPage Novex 4–12% BIS/Tris gel (ThermoFisher Scientific), along with a molecular weight marker to confirm blot transfer and 3 μL of Magic Mark™ molecular weight marker (ThermoFisher Scientific) to estimate protein size. Protein was transferred to a PVDF membrane using the iblot 2 Dry Blotting System (Thermo Fisher Scientific). Membranes were blocked in 5% skim milk powder in 1 × TBST for 1 h at room temperature. The primary anti-FUS/TLS antibody (Santa Cruz Biotechnology, sc-47711) was added at a 1:1000 dilution and incubated overnight on a plate rocker at 4 °C. Membranes were then washed in 1× TBST for 3 × 20 min and then incubated in a goat anti-rabbit HRP-labelled secondary antibody (Dako, Santa Clara, CA, USA) at a 1 in 3000 dilution for 1 h. Membranes were washed in TBST for 3 × 20 min before signals were detected using the Immobilon Western Chemiluminescent HRP Substrate (Merck, Darmstadt, Germany). Western blot images were taken on a Vilber Lourmat FusionFX system using Fusion software version 17.03. Total protein in each sample was measured by washing the membrane in water, then staining for 5 min in a stain containing 0.025% *w*/*v* Coomassie blue, 40% methanol, 7% glacial acetic acid and water. Membranes were destained in a solution containing 50% methanol, 7% glacial acetic acid, and water for 5 min before total protein was imaged. Densitometry analysis of images was undertaken using ImageJ software version 2.1.0/1.53j with FUS levels normalized to total protein.

### 4.6. Immunocytochemistry

Cells were transfected and incubated on 12-well coverslips for 24 h. Cells were washed with PBS, then fixed in 1:1 acetone/methanol on ice for 4 min before freezing at −80 °C until staining. Coverslips were thawed and the cell membranes permeabilized in 1% Triton-X-100 in PBS for 5 min. Cells were washed in PBS for 5 min before being blocked for 30 min using 10% filtered goat serum in 0.05% tween-20 in PBS. Cells were immunostained with anti-FUS/TLS antibody (Santa Cruz Biotechnology, sc-47711), anti-SFPQ antibody (Abcam, Ab38148) and anti-β-III Tubulin (Novus Biologicals, NB100-1612) antibodies diluted in in 0.05% tween-20 in PBS for 1 h at room temperature. Cells were washed in three 5 min wash steps in 0.02% Triton-X-100 in PBS and secondary antibodies applied, goat-anti mouse Alexa Fluor 568, goat-anti rabbit Alexa Flour 680, and goat-anti chicken Alexa Fluor 488 (Thermo Fisher Scientific, A-11004, A27042, 35518) diluted in 0.05% tween-20 in PBS, for 1 h at room temperature. Nuclei were stained with Hoechst 33342 (Sigma-Aldrich), 1 mg/mL, diluted 1:125 for 5 min. Coverslips were mounted using ProLong™ Gold antifade mountant (Thermo Fisher Scientific). Images were captured using an ECHO revolve microscope and software using the 20× objective. Several fields of view were captured that were selected while visualizing the Hoechst stain with exposure parameters remaining constant between images and coverslips. Images were blinded before quantification. Each cell was compared to example cells and categorized as having SFPQ fibrils, aggregation, mild aggregation, or dispersed SFPQ.

### 4.7. cDNA Synthesis and qPCR

cDNA was synthesized from RNA using random primers and the Superscript IV first-strand synthesis system (Life Technologies, ThermoFisher Scientific, Scoresby, VIC, Australia) as per the manufacturer’s instructions. cDNA was diluted 1 in 5 before use in qPCRs. Prime time gene expression master mix and qPCR assays (Hs.PT.58.835567, Hs.PT.58.164145, Hs.PT.58.4795767, Hs.PT.58.22962649, Hs.PT.58.26101059, Hs.PT.58.38763048, Hs.PT.56a.3116792, Hs.PT.58.4870725, Hs.PT.58.4756064, Hs.PT.58.26571837, Hs.PT.58v.39858774) containing primers and probes were purchased from Integrated DNA Technologies Australia (Melbourne, VIC, Australia). Reactions were set up in triplicate in 384-well plates. The plates were run on a CFX384 TouchTM Real-Time PCR detection system (Bio-Rad Laboratories). The PCR reaction was performed using the following conditions: 95 °C for 3 min followed by 40 cycles of 95 °C for 15 s and 60 °C for 1 min. TBP expression served as a housekeeping control. Normalized gene expression values against TBP were obtained using the Pfaffl method [72], incorporating primer efficiencies on each plate run.

## 5. Patents

Compositions and Methods for Treating Diseases Associated with Pathogenic FUS Variants, PCT/AU2023/050067.

## Figures and Tables

**Table 1:** Percentage of the 1000 genome populations heterozygous for the selected *FUS* CVs.

Populations	CV1	CV2	CV1 and/or CV2	CV1 and CV2
European	42.9	45.9	56.5	32.3
American	45.8	49.9	57.9	37.8
Asian	41.1	41.4	42.6	39.9
African	5.3	34.2	38.8	0.7

**Table 2:** AO sequences and chemistry.

AO Name	Gapmer Configuration	Complementary to	Sequence (5′-3′)
CV1a1	20 (5-10-5)	CV1 Allele 1	TCTGGCCATATCCTGAAGTG
CV1a1-1	20 (6-8-6)	CV1 Allele 1	TCTGGCCATATCCTGAAGTG
CV1a1-2	20 (7-6-7)	CV1 Allele 1	TCTGGCCATATCCTGAAGTG
CV1a1-3	18 (5-8-5)	CV1 Allele 1	CTGGCCATATCCTGAAGT
CV1a1-4	16 (5-6-5)	CV1 Allele 1	TGGCCATATCCTGAAG
CV1a2	20 (5-10-5)	CV1 Allele 2	TCTGGCCATAGCCTGAAGTG
CV1a2-1	20 (6-8-6)	CV1 Allele 2	TCTGGCCATAGCCTGAAGTG
CV1a2-2	20 (7-6-7)	CV1 Allele 2	TCTGGCCATAGCCTGAAGTG
CV1a2-3	18 (5-8-5)	CV1 Allele 2	CTGGCCATAGCCTGAAGT
CV1a2-4	16 (5-6-5)	CV1 Allele 2	TGGCCATAGCCTGAAG
CV1a2b	20 (5-10-5)	CV1 Allele 2	GCCATAGCCTGAAGTGTCCG
CV2a1	20 (5-10-5)	CV2 Allele 1	CATAGCCAGGGTAGGAGGAC
CV2a1-1	20 (6-8-6)	CV2 Allele 1	CATAGCCAGGGTAGGAGGAC
CV2a1-2	20 (7-6-7)	CV2 Allele 1	CATAGCCAGGGTAGGAGGAC
CV2a1-3	18 (5-8-5)	CV2 Allele 1	ATAGCCAGGGTAGGAGGA
CV2a1-4	16 (5-6-5)	CV2 Allele 1	TAGCCAGGGTAGGAGG
CV2a2	20 (5-10-5)	CV2 Allele 2	CATAGCCAGGATAGGAGGAC
CV2a2-1	20 (6-8-6)	CV2 Allele 2	CATAGCCAGGATAGGAGGAC
CV2a2-2	20 (7-6-7)	CV2 Allele 2	CATAGCCAGGATAGGAGGAC
CV2a2-3	18 (5-8-5)	CV2 Allele 2	ATAGCCAGGATAGGAGGA
CV2a2-4	16 (5-6-5)	CV2 Allele 2	TAGCCAGGATAGGAGG
Scramble Control	20 (5-10-5)	None	ACCTTATCCAATAGCGCCTC
Selectivity Control	20 (5-10-5)	Both alleles	CCACTGTAACTCTGCTGTCC
Ulefnersen mimic	20 (5-10-5)	Both alleles	GC*A*A*T*GTCACCTTTCA*T*ACC

Blue letters: morpholino 3′-thiophosphoramidate nucleotide (TMO) or 2′methoxyethyl (MOE), black letters: 2′-deoxynucleotide, green letters: 2′-deoxynucleoside for the TMO-modified AOs. There are PS linkages throughout except in the Ulefnersen mimic, where * indicates a phosphodiester internucleoside linkage. Cytosines are methylated at the 5′ position for all MOE-modified AOs. Underlined base indicates the position complementary to CV1 or CV2.

## Data Availability

Data are available from the corresponding author upon request.

## Supplementary Materials

The following supporting information can be downloaded at: https://www.mdpi.com/article/10.3390/ijms25158495/s1.

## References

[1] Rowland L.P., Shneider N.A. (2001). Amyotrophic Lateral Sclerosis. N. Engl. J. Med..

[2] Kirby J., Al Sultan A., Waller R., Heath P. (2016). The genetics of amyotrophic lateral sclerosis: Current insights. Degener. Neurol. Neuromuscul. Dis..

[3] Zou Z.-Y., Cui L.-Y., Sun Q., Li X.-G., Liu M.-S., Xu Y., Zhou Y., Yang X.-Z. (2013). De novo FUS gene mutations are associated with juvenile-onset sporadic amyotrophic lateral sclerosis in China. Neurobiol. Aging.

[4] Gromicho M., Oliveira Santos M., Pinto A., Pronto-Laborinho A., De Carvalho M. (2017). Young-onset rapidly progressive ALS associated with heterozygous FUS mutation. Amyotroph. Lateral Scler. Front. Degener..

[5] Hübers A., Just W., Rosenbohm A., Müller K., Marroquin N., Goebel I., Högel J., Thiele H., Altmüller J., Nürnberg P. (2015). De novo FUS mutations are the most frequent genetic cause in early-onset German ALS patients. Neurobiol. Aging.

[6] Hübers A., Volk A., Just W., Rosenbohm A., Bierbaumer N., Kathrin M., Nicolai M., Ingrid G., Josef H., Janine A. (2015). V42. De novo mutations in the FUS gene are a frequent cause of sporadic ALS in very young patients. Clin. Neurophysiol..

[7] Zou Z.-Y., Zhou Z.-R., Che C.-H., Liu C.-Y., He R.-L., Huang H.-P. (2017). Genetic epidemiology of amyotrophic lateral sclerosis: A systematic review and meta-analysis. J. Neurol. Neurosurg. Psychiatry.

[8] Lattante S., Rouleau G.A., Kabashi E. (2013). TARDBP and FUS Mutations Associated with Amyotrophic Lateral Sclerosis: Summary and Update. Hum. Mutat..

[9] Vance C., Scotter E.L., Nishimura A.L., Troakes C., Mitchell J.C., Kathe C., Urwin H., Manser C., Miller C.C., Hortobágyi T. (2013). ALS mutant FUS disrupts nuclear localization and sequesters wild-type FUS within cytoplasmic stress granules. Hum. Mol. Genet..

[10] Niu C., Zhang J., Gao F., Yang L., Jia M., Zhu H., Gong W. (2012). FUS-NLS/Transportin 1 complex structure provides insights into the nuclear targeting mechanism of FUS and the implications in ALS. PLoS ONE.

[11] Shang Y., Huang E.J. (2016). Mechanisms of FUS Mutations in Familial Amyotrophic Lateral Sclerosis. Brain Res..

[12] Nomura T., Watanabe S., Kaneko K., Yamanaka K., Nukina N., Furukawa Y. (2014). Intranuclear aggregation of mutant FUS/TLS as a molecular pathomechanism of amyotrophic lateral sclerosis. J. Biol. Chem..

[13] Van Daele S.H., Masrori P., Van Damme P., Van Den Bosch L. (2024). The sense of antisense therapies in ALS. Trends Mol. Med..

[14] Hyjek M., Figiel M., Nowotny M. (2019). RNases H: Structure and mechanism. DNA Repair.

[15] Stein C.A., Subasinghe C., Shinozuka K., Cohen J.S. (1988). Physicochemical properties of phosphorothioate oligodeoxynucleotides. Nucleic Acids Res..

[16] Crooke S.T., Vickers T.A., Liang X.H. (2020). Phosphorothioate modified oligonucleotide-protein interactions. Nucleic Acids Res..

[17] Pollak A.J., Zhao L., Vickers T.A., Huggins I.J., Liang X.H., Crooke S.T. (2022). Insights into innate immune activation via PS-ASO-protein-TLR9 interactions. Nucleic Acids Res..

[18] Collotta D., Bertocchi I., Chiapello E., Collino M. (2023). Antisense oligonucleotides: A novel Frontier in pharmacological strategy. Front. Pharmacol..

[19] Hill A.C., Hall J. (2023). The MOE Modification of RNA: Origins and Widescale Impact on the Oligonucleotide Therapeutics Field. Helv. Chim. Acta.

[20] Summerton J., Weller D. (1997). Morpholino antisense oligomers: Design, preparation, and properties. Antisense Nucleic Acid Drug Dev..

[21] Hudziak R.M., Barofsky E., Barofsky D.F., Weller D.L., Huang S.B., Weller D.D. (1996). Resistance of morpholino phosphorodiamidate oligomers to enzymatic degradation. Antisense Nucleic Acid Drug Dev..

[22] Mendell J., Powers J., Duda P., Eliopoulos H. (2016). Clinical safety of eteplirsen, a phosphorodiamidate morpholino oligomer (PMO), in Duchenne muscular dystrophy (DMD) patients amenable to skipping exon 51 of the DMD gene. Neuromuscul. Disord..

[23] Crooke S.T., Liang X.-H., Baker B.F., Crooke R.M. (2021). Antisense technology: A review. J. Biol. Chem..

[24] Langner H.K., Jastrzebska K., Caruthers M.H. (2020). Synthesis and Characterization of Thiophosphoramidate Morpholino Oligonucleotides and Chimeras. J. Am. Chem. Soc..

[25] Korobeynikov V.A., Lyashchenko A.K., Blanco-Redondo B., Jafar-Nejad P., Shneider N.A. (2022). Antisense oligonucleotide silencing of FUS expression as a therapeutic approach in amyotrophic lateral sclerosis. Nat. Med..

[26] Clarke L., Fairley S., Zheng-Bradley X., Streeter I., Perry E., Lowy E., Tassé A.-M., Flicek P. (2016). The international Genome sample resource (IGSR): A worldwide collection of genome variation incorporating the 1000 Genomes Project data. Nucleic Acids Res..

[27] Flynn L.L., Li R., Pitout I.L., Larcher L.M., Cooper J.A., Aung-Htut M.T.H., Hubbard A., Griffiths L., Bond C.S., Wilton S.D. (2022). Single stranded fully modified-phosphorothioate oligonucleotides rapidly induce structured nuclear inclusions and global alterations to the transcriptome in vitro. Front. Genet..

[28] Qiu H., Lee S., Shang Y., Wang W.-Y., Au K.F., Kamiya S., Barmada S.J., Finkbeiner S., Lui H., Carlton C.E. (2014). ALS-associated mutation FUS-R521C causes DNA damage and RNA splicing defects. J. Clin. Investig..

[29] Yasuhara H., Yoshida T., Sasaki K., Obika S., Inoue T. (2022). Reduction of Off-Target Effects of Gapmer Antisense Oligonucleotides by Oligonucleotide Extension. Mol. Diagn. Ther..

[30] López-Erauskin J., Tadokoro T., Baughn M.W., Myers B., McAlonis-Downes M., Chillon-Marinas C., Asiaban J.N., Artates J., Bui A.T., Vetto A.P. (2018). ALS/FTD-Linked Mutation in FUS Suppresses Intra-axonal Protein Synthesis and Drives Disease Without Nuclear Loss-of-Function of FUS. Neuron.

[31] Scekic-Zahirovic J., Sendscheid O., El Oussini H., Jambeau M., Sun Y., Mersmann S., Wagner M., Dieterlé S., Sinniger J., Dirrig-Grosch S. (2016). Toxic gain of function from mutant FUS protein is crucial to trigger cell autonomous motor neuron loss. EMBO J..

[32] Sharma A., Lyashchenko A.K., Lu L., Nasrabady S.E., Elmaleh M., Mendelsohn M., Nemes A., Tapia J.C., Mentis G.Z., Shneider N.A. (2016). ALS-associated mutant FUS induces selective motor neuron degeneration through toxic gain of function. Nat. Commun..

[33] Ishigaki S., Sobue G. (2018). Importance of Functional Loss of FUS in FTLD/ALS. Front. Mol. Biosci..

[34] Szewczyk B., Günther R., Japtok J., Frech M.J., Naumann M., Lee H.O., Hermann A. (2023). FUS ALS neurons activate major stress pathways and reduce translation as an early protective mechanism against neurodegeneration. Cell Rep..

[35] Ratti A., Buratti E. (2016). Physiological functions and pathobiology of TDP-43 and FUS/TLS proteins. J. Neurochem..

[36] Lagier-Tourenne C., Polymenidou M., Hutt K.R., Vu A.Q., Baughn M., Huelga S.C., Clutario K.M., Ling S.-C., Liang T.Y., Mazur C. (2012). Divergent roles of ALS-linked proteins FUS/TLS and TDP-43 intersect in processing long pre-mRNAs. Nat. Neurosci..

[37] Colombrita C., Onesto E., Buratti E., de la Grange P., Gumina V., Baralle F.E., Silani V., Ratti A. (2015). From transcriptomic to protein level changes in TDP-43 and FUS loss-of-function cell models. Biochim. Biophys. Acta (BBA)—Gene Regul. Mech..

[38] Reber S., Stettler J., Filosa G., Colombo M., Jutzi D., Lenzken S.C., Schweingruber C., Bruggmann R., Bachi A., Barabino S.M. (2016). Minor intron splicing is regulated by FUS and affected by ALS-associated FUS mutants. EMBO J..

[39] Ishigaki S., Fujioka Y., Okada Y., Riku Y., Udagawa T., Honda D., Yokoi S., Endo K., Ikenaka K., Takagi S. (2017). Altered Tau Isoform Ratio Caused by Loss of FUS and SFPQ Function Leads to FTLD-like Phenotypes. Cell Rep..

[40] Orozco D., Tahirovic S., Rentzsch K., Schwenk B.M., Haass C., Edbauer D. (2012). Loss of fused in sarcoma (FUS) promotes pathological Tau splicing. EMBO Rep..

[41] Rösler T.W., Tayaranian Marvian A., Brendel M., Nykänen N.P., Höllerhage M., Schwarz S.C., Hopfner F., Koeglsperger T., Respondek G., Schweyer K. (2019). Four-repeat tauopathies. Prog. Neurobiol..

[42] Ishigaki S., Riku Y., Fujioka Y., Endo K., Iwade N., Kawai K., Ishibashi M., Yokoi S., Katsuno M., Watanabe H. (2020). Aberrant interaction between FUS and SFPQ in neurons in a wide range of FTLD spectrum diseases. Brain.

[43] Baechtold H., Kuroda M., Sok J., Ron D., Lopez B.S., Akhmedov A.T. (1999). Human 75-kDa DNA-pairing Protein Is Identical to the Pro-oncoprotein TLS/FUS and Is Able to Promote D-loop Formation. J. Biol. Chem..

[44] Mastrocola A.S., Kim S.H., Trinh A.T., Rodenkirch L.A., Tibbetts R.S. (2013). The RNA-binding protein fused in sarcoma (FUS) functions downstream of poly(ADP-ribose) polymerase (PARP) in response to DNA damage. J. Biol. Chem..

[45] Wang W.-Y., Pan L., Su S.C., Quinn E.J., Sasaki M., Jimenez J.C., Mackenzie I.R.A., Huang E.J., Tsai L.-H. (2013). Interaction of FUS and HDAC1 regulates DNA damage response and repair in neurons. Nat. Neurosci..

[46] Sukhanova M.V., Singatulina A.S., Pastré D., Lavrik O.I. (2020). Fused in Sarcoma (FUS) in DNA Repair: Tango with Poly(ADP-ribose) Polymerase 1 and Compartmentalisation of Damaged DNA. Int. J. Mol. Sci..

[47] Levone B.R., Lenzken S.C., Antonaci M., Maiser A., Rapp A., Conte F., Reber S., Mechtersheimer J., Ronchi A.E., Mühlemann O. (2021). FUS-dependent liquid–liquid phase separation is important for DNA repair initiation. J. Cell Biol..

[48] Wang H., Guo W., Mitra J., Hegde P.M., Vandoorne T., Eckelmann B.J., Mitra S., Tomkinson A.E., Van Den Bosch L., Hegde M.L. (2018). Mutant FUS causes DNA ligation defects to inhibit oxidative damage repair in Amyotrophic Lateral Sclerosis. Nat. Commun..

[49] Sama R.R.K., Ward C.L., Kaushansky L.J., Lemay N., Ishigaki S., Urano F., Bosco D.A. (2013). FUS/TLS assembles into stress granules and is a prosurvival factor during hyperosmolar stress. J. Cell. Physiol..

[50] Lenzi J., De Santis R., De Turris V., Morlando M., Laneve P., Calvo A., Caliendo V., Chiò A., Rosa A., Bozzoni I. (2015). ALS mutant FUS proteins are recruited into stress granules in induced pluripotent stem cell-derived motoneurons. DMM Dis. Models Mech..

[51] Wolozin B., Ivanov P. (2019). Stress granules and neurodegeneration. Nat. Rev. Neurosci..

[52] Fox A.H., Nakagawa S., Hirose T., Bond C.S. (2018). Paraspeckles: Where Long Noncoding RNA Meets Phase Separation. Trends Biochem. Sci..

[53] Hennig S., Kong G., Mannen T., Sadowska A., Kobelke S., Blythe A., Knott G.J., Iyer K.S., Ho D., Newcombe E.A. (2015). Prion-like domains in RNA binding proteins are essential for building subnuclear paraspeckles. J. Cell Biol..

[54] An H., Skelt L., Notaro A., Highley J.R., Fox A.H., La Bella V., Buchman V.L., Shelkovnikova T.A. (2019). ALS-linked FUS mutations confer loss and gain of function in the nucleus by promoting excessive formation of dysfunctional paraspeckles. Acta Neuropathol. Commun..

[55] Shelkovnikova T.A., Robinson H.K., Troakes C., Ninkina N., Buchman V.L. (2013). Compromised paraspeckle formation as a pathogenic factor in FUSopathies. Hum. Mol. Genet..

[56] Le B.T., Paul S., Jastrzebska K., Langer H., Caruthers M.H., Veedu R.N. (2022). Thiomorpholino oligonucleotides as a robust class of next generation platforms for alternate mRNA splicing. Proc. Natl. Acad. Sci. USA.

[57] Monia B.P., Johnston J.F., Ecker D.J., Zounes M.A., Lima W.F., Freier S.M. (1992). Selective inhibition of mutant Ha-ras mRNA expression by antisense oligonucleotides. J. Biol. Chem..

[58] Rukov J.L., Hagedorn P.H., Høy I.B., Feng Y., Lindow M., Vinther J. (2015). Dissecting the target specificity of RNase H recruiting oligonucleotides using massively parallel reporter analysis of short RNA motifs. Nucleic Acids Res..

[59] Lima W.F., Rose J.B., Nichols J.G., Wu H., Migawa M.T., Wyrzykiewicz T.K., Vasquez G., Swayze E.E., Crooke S.T. (2007). The Positional Influence of the Helical Geometry of the Heteroduplex Substrate on Human RNase H1 Catalysis. Mol. Pharmacol..

[60] Giles R.V., Ruddell C.J., Spiller D.G., Green J.A., Tidd D.M. (1995). Single base discrimination for ribonuclease H-dependent antisense effects within intact human leukaemia cells. Nucleic Acids Res..

[61] Magner D., Biala E., Lisowiec-Wachnicka J., Kierzek E., Kierzek R. (2015). A Tandem Oligonucleotide Approach for SNP-Selective RNA Degradation Using Modified Antisense Oligonucleotides. PLoS ONE.

[62] Kiełpiński Ł.J., Hagedorn P.H., Lindow M., Vinther J. (2017). RNase H sequence preferences influence antisense oligonucleotide efficiency. Nucleic Acids Res..

[63] Wu H., Lima W.F., Zhang H., Fan A., Sun H., Crooke S.T. (2004). Determination of the Role of the Human RNase H1 in the Pharmacology of DNA-like Antisense Drugs. J. Biol. Chem..

[64] Crooke S.T. (2017). Molecular Mechanisms of Antisense Oligonucleotides. Nucleic Acid Ther..

[65] Wu H., Lima W.F., Crooke S.T. (1999). Properties of cloned and expressed human RNase H1. J. Biol. Chem..

[66] Stanton R., Sciabola S., Salatto C., Weng Y., Moshinsky D., Little J., Walters E., Kreeger J., DiMattia D., Chen T. (2012). Chemical Modification Study of Antisense Gapmers. Nucleic Acid Ther..

[67] Straarup E.M., Fisker N., Hedtjarn M., Lindholm M.W., Rosenbohm C., Aarup V., Hansen H.F., Ørum H., Hansen J.B., Koch T. (2010). Short locked nucleic acid antisense oligonucleotides potently reduce apolipoprotein B mRNA and serum cholesterol in mice and non-human primates. Nucleic Acids Res..

[68] Pedersen L., Hagedorn P.H., Lindholm M.W., Lindow M. (2014). A Kinetic Model Explains Why Shorter and Less Affine Enzyme-recruiting Oligonucleotides Can Be More Potent. Mol. Ther. Nucleic. Acids.

[69] Herschlag D. (1991). Implications of ribozyme kinetics for targeting the cleavage of specific RNA molecules in vivo: More isn’t always better. Proc. Natl. Acad. Sci. USA.

[70] Magner D., Biala E., Lisowiec-wachnicka J., Kierzek R. (2017). Influence of mismatched and bulged nucleotides on SNP-preferential RNase H cleavage of RNA-antisense gapmer heteroduplexes. Sci. Rep..

[71] Xiao X., Li M., Ye Z., He X., Wei J., Zha Y. (2024). FUS gene mutation in amyotrophic lateral sclerosis: A new case report and systematic review. Amyotroph Lateral Scler Front. Degener.

[72] Pfaffl M.W. (2001). A new mathematical model for relative quantification in real-time RT-PCR. Nucleic Acids Res..

