# Supplementary material for: Allele-Selective Thiomorpholino Antisense Oligonucleotides as a Therapeutic Approach for Fused-in-Sarcoma Amyotrophic Lateral Sclerosis
Source: Int J Mol Sci. 2024 Aug 3;25(15):8495. doi: 10.3390/ijms25158495 (PMC11312655; doi:10.3390/ijms25158495)
Supplement: Supplementary file 1 [file ijms-25-08495-s001.zip › ijms-3104283-supplementary.pdf]

Supplementary Table S1: qPCR results for off-target genes 72 hrs after transfection with CV1 targeted AOs showing mean expression and 95% confidence interval compared to transfection control treated cells (α= 0.05).

Gene	AO	20(5-10-5)	20(6-8-6)	20(7-6-7)	18(5-8-5)	16(5-6-5)
REV3L	CV1a1	0.55 [0.006, 1.09)	**0.38 [0.05, 0.71]**	0.63 [0.23, 1.03]	**0.28 [0.10, 0.46]**	0.89 [0.36,1.41]
EBPL	CV1a2	**0.17 [0.06, 0.28]**	**0.21 [0.05, 0.36]**	1.14 [0.85, 1.43]	**0.22 [0.16, 0.33]**	1.18 [0.73, 1.63]
FNBP1L	CV1a1	1.52 [0.97, 2.10]	1.45 [1.11, 1.79]	1.34 [0.99, 1.70]	0.92 [0.51, 1.32]	1.33 [0.93, 1.72]
RIC8B	CV1a2	0.97 [0.91, 1.02]	0.98 [0.71, 1.24]	1.05 [0.66, 1.45]	**0.88 [0.85, 0.92]**	1.04 [0.72, 1.36]
REV3L	CV1a2	0.83 [0.42, 1.25]	0.73 [0.47, 1.00]	1.08 [0.90, 1.26]	0.76 [0.12, 1.40]	1.01 [0.62, 1.39]
DHCR7	CV1a2	1.00 [0.02, 1.98]	0.46 [0.00, 1.30]	1.30 [0.00, 2.91]	**0.38 [0.15, 0.61]**	1.13 [0.77, 1.49]
DENND1B	CV1a2	0.71 [0.28, 1.13]	0.59 [0.17, 1.02]	0.94 [0.35, 1.52]	**0.34 [0.01, 0.67]**	1.08 [0.32, 1.84]
EBPL	CV1a1	1.04 [0.40, 1.68]	1.53 [0.93, 2.13]	1.05 [0.81, 1.29]	1.14 [0.74, 1.53]	1.02 [0.80, 1.25]
FBN1	CV1a1	**0.44 [0.27, 0.61]**	**0.37 [0.20, 0.53]**	0.83 [0.54, 1.13]	**0.25 [0.13, 0.38]**	0.95 [0.68, 1.23]
ASMTL	CV1a1	0.89 [0.00, 1.81]	0.79 [0.29, 1.28]	0.72 [0.41, 1.03]	0.74 [0.30, 1.18]	0.71 [0.28, 1.15]
ACVRL1	CV1a1	0.96 [0.20, 1.73]	1.03 [0.60, 1.46]	1.32 [0.84, 1.80]	0.56 [0.00, 1.18]	1.29 [0.40, 2.19]
FNBP1L	CV1a2	1.21 [0.97, 1.45]	0.72 [0.0, 1.46]	1.08 [0.53, 1.62]	0.98 [0.50, 1.46]	1.50 [0.88, 2.12]
RIC8B	CV1a1	**0.68 [0.61, 0.75]**	**0.80 [0.69, 0.90]**	**0.86 [0.82, 0.90]**	**0.52 [0.37, 0.67]**	1.00 [0.94, 1.05]
DHCR7	CV1a1	1.18 [0.40, 1.96]	1.50 [0.58, 2.43]	1.71 [0.54, 2.88]	1.41 [0.01, 2.82]	1.61 [0.62, 2.60]
DENND1B	CV1a1	0.73 [0.00, 1.64]	**0.51 [0.06, 0.96]**	**0.69 [0.43, 0.95]**	**0.51 [0.37, 0.65]**	0.78 [0.37, 1.18]
FBN1	CV1a2	**0.75 [0.61, 0.90]**	0.67 [0.06, 1.28]	0.99 [0.63, 1.35]	**0.53 [0.36, 0.70]**	1.19 [0.45, 1.93]
TOP2B	CV1a1	0.80 [0.00, 2.68]	0.77 [0.23, 1.32]	0.92 [0.15, 1.69]	0.82 [0.46, 1.17]	0.94 [0.21, 1.66]
TOP2B	CV1a2	0.85 [0.25, 1.44]	0.76 [0.32, 1.20]	1.28 [0.00, 2.56]	0.72 [0.07, 1.37]	1.30 [0.02, 2.58]
ACVRL1	CV1a2	1.05 [0.30, 1.79]	0.57 [0.00, 1.24]	0.99 [0.83, 1.15]	**0.45 [0.15, 0.75]**	0.98 [0.25, 1.71]
ASMTL	CV1a2	0.91 [0.40, 1.41]	1.21 [0.11, 1.31]	1.19 [0.63, 1.74]	1.24 [0.54, 1.94]	1.13 [0.51, 1.75]

Supplementary Table S2: qPCR results for off-target genes 72 hrs after transfection with control AOs showing mean and 95% confidence interval compared to transfection control treated cells (α= 0.05).

Gene	CV2a1	CV2a2	Ulef mim
REV3L	0.93 [0.33, 1.54]	0.92 [0.40, 1.43]	1.48 [0.75, 2.21]
EBPL	1.08 [0.69, 1.46]	1.19 [0.39, 1.99]	1.54 [0.62, 2.45]
FNBP1L	1.21 [0.41, 2.01]	1.21 [0.20, 2.21]	1.77 [0.82, 2.71]
RIC8B	0.87 [0.71, 1.02]	0.83 [0.73, 0.93]	**0.84 [0.73, 0.96]**
REV3L	0.93 [0.33, 1.54]	0.92 [0.40, 1.43]	1.48 [0.75, 2.21]
DHCR7	1.12 [0.55, 1.69]	1.25 [0.78, 1.72]	1.38 [0.00, 2.85]
DENND1B	1.17 [0.72, 1.63]	1.32 [0.60, 2.04]	0.97 [0.48, 1.46]
EBPL	1.08 [0.69, 1.46]	1.19 [0.39, 1.99]	1.54 [0.62, 2.45]
FBN1	1.08 [0.46, 1.71]	0.97 [0.68, 1.26]	0.93 [0.70, 1.16]
ASMTL	1.03 [0.26, 1.80]	1.16 [0.20, 2.11]	0.74 [0.02, 1.47]
ACVRL1	1.08 [0.48, 1.67]	0.88 [0.28, 1.48]	1.07 [0.13. 2.02]
FNBP1L	1.21 [0.41, 2.01]	1.21 [0.20, 2.21]	1.77 [0.82, 2.71]
RIC8B	0.87 [0.71, 1.02]	0.83 [0.73, 0.93]	0.84 [0.73, 0.96]
DHCR7	1.12 [0.55, 1.69]	1.25 [0.78, 1.72]	1.38 [0.00, 2.85]
DENND1B	1.17 [0.72, 1.63]	1.32 [0.60, 2.04]	0.97 [0.48, 1.46]
FBN1	1.08 [0.46, 1.71]	0.97 [0.68, 1.26]	0.93 [0.70, 1.16]
TOP2B	1.39 [0.53, 2.25]	1.42 [0.30, 2.54]	0.99 [0.00, 2.06]
TOP2B	1.39 [0.53, 2.25]	1.42 [0.30, 2.54]	0.99 [0.00, 2.06]
ACVRL1	1.08 [0.48, 1.67]	0.88 [0.28, 1.48]	1.07 [0.13. 2.02]
ASMTL	1.03 [0.26, 1.80]	1.16 [0.20, 2.11]	0.74 [0.02, 1.47]
